# Acute minocycline administration reduces brain injury and improves long-term functional outcomes after delayed hypoxemia following traumatic brain injury

**DOI:** 10.1186/s40478-022-01310-1

**Published:** 2022-01-28

**Authors:** Marta Celorrio, Kirill Shumilov, Camryn Payne, Sangeetha Vadivelu, Stuart H. Friess

**Affiliations:** Division of Critical Care Medicine, Department of Pediatrics, Washington University in St. Louis School of Medicine, 660 South Euclid Avenue, St. Louis, MO 63110 USA

**Keywords:** Traumatic brain injury, Hypoxemia, Minocycline, Neuroprotection, Secondary injury, Pre-clinical trial

## Abstract

Clinical trials of therapeutics for traumatic brain injury (TBI) demonstrating preclinical efficacy for TBI have failed to replicate these results in humans, in part due to the absence of clinically feasible therapeutic windows for administration. Minocycline, an inhibitor of microglial activation, has been shown to be neuroprotective when administered early after experimental TBI but detrimental when administered chronically to human TBI survivors. Rather than focusing on the rescue of primary injury with early administration of therapeutics which may not be clinically feasible, we hypothesized that minocycline administered at a clinically feasible time point (24 h after injury) would be neuroprotective in a model of TBI plus delayed hypoxemia. We first explored several different regimens of minocycline dosing with the initial dose 24 h after injury and 2 h prior to hypoxemia, utilizing short-term neuropathology to select the most promising candidate. We found that a short course of minocycline reduced acute microglial activation, monocyte infiltration and hippocampal neuronal loss at 1 week post injury. We then conducted a preclinical trial to assess the long-term efficacy of a short course of minocycline finding reductions in hippocampal neurodegeneration and synapse loss, preservation of white matter myelination, and improvements in fear memory performance at 6 months after injury. Timing in relation to injury and duration of minocycline treatment and its impact on neuroinflammatory response may be responsible for extensive neuroprotection observed in our studies.

**Supplementary Information:**

The online version contains supplementary material available at 10.1186/s40478-022-01310-1.

## Introduction

Approximately 1.7 million people experience traumatic brain injury (TBI) each year and over 5 million face TBI-related disabilities [34]. Optimization of physiologic parameters and minimization of secondary insults such as hypotension, hypoxia, intracranial hypertension, and excitotoxicity are the primary goals of care after moderate and severe TBI [6, 26]. Neuroprotective agents in experimental TBI have shown promise when administered before or very early after injury [16, 30, 48]. However, clinical trials of therapeutics demonstrating preclinical efficacy for TBI have failed to replicate these results in humans, in part due to the absence of clinically feasible therapeutic windows for administration of neuroprotective agents [10, 15, 25, 38, 44, 54].

Minocycline is a broad-spectrum tetracycline antibiotic, which has been studied as a neuroprotective agent in TBI [32, 43, 46]. Although minocycline is proposed to have pleiotropic effects on the brain, the main mechanism of action for neuroprotection is thought to be suppression of microglial activation after TBI [43]. Preclinical investigations on the efficacy of minocycline have utilized short temporal windows of administration (within one hour) after injury reducing translatability of these findings to the clinical setting [20, 35, 42]. A recent clinical trial of minocycline for chronic TBI demonstrated a reduction in chronic microglial activation but an exacerbation of neuronal degeneration in patients receiving minocycline for 12 weeks highlighting that neuroprotective benefits of minocycline may be dependent on timing of administration [39]. Furthermore, recent evidence suggests that timing and duration of microglial modulation in experimental TBI may impact injury severity and recovery [23, 52].

We have developed a mouse model of TBI with delayed secondary hypoxemia to incorporate a common secondary insult encountered by TBI patients in the intensive care setting [31]. We have previously demonstrated that delayed normocarbic hypoxemia 24 h after experimental TBI exacerbated injury and behavioral deficits up to 6 months after injury [14, 31]. Rather than focusing on the rescue of primary injury with early administration of therapeutics which may not be clinically feasible, we hypothesized that minocycline administered at a clinically feasible time point (24 h after injury) would be neuroprotective in a model of TBI plus delayed hypoxemia. Unlike the treatment of primary TBI in the field, TBI patients in the intensive care setting provide an opportunity for rapid administration of therapeutics with narrow temporal windows of efficacy.

In this preclinical trial, we first explored several different regimens of minocycline dosing with the initial dose 24 h after injury and 2 h prior to hypoxemia, utilizing short-term neuropathology to select the most promising candidate. We then conducted a preclinical trial to assess the long-term efficacy of a short course of minocycline finding reductions in hippocampal neurodegeneration and synapse loss, preservation of white matter myelination, and improvements in fear memory performance at a clinically relevant time point (6 months after injury).

## Material and methods

### Traumatic brain injury and delayed hypoxemia

All procedures were approved by the Washington University Animal Studies Committee and are consistent with the National Institutes of Health guidelines for the care and use of animals. Animals were housed 5/cage and had free access to water and food with 12-h light/dark cycle. C57BL/6 J 8-week old male mice (Jackson Laboratory, Bar Harbor, ME) were used in the initial dose response study. For subsequent studies with the selected dose, C57BL/6 J 8-week old male and female mice (Jackson Laboratory, Bar Harbor, ME) were used. Mice were anesthetized with 5% isoflurane at induction, followed by maintenance at 2% isoflurane for the duration of the procedure. Buprenorphine sustained release (0.5 mg/kg subcutaneously, Zoopharm, Windsor, CO) was administered prior to scalp incision. The head was shaved and ear bars were used to stabilize the head within the stereotaxic frame (MyNeurolab, St. Louis, MO). Then, a single 5-mm craniectomy was performed by an electric drill on the left lateral side of the skull centered 2.7 mm lateral from the midline and 3 mm anterior to lambda. For flow cytometry experiments and long-term behavioral studies, animals were randomized to sham or injury after craniectomy using a computer-generated numbers randomization. For animals undergoing controlled cortical impact (CCI), the 3-mm electromagnetic impactor tip was then aligned with the craniectomy site at 1.2 mm left of midline, 1.5 mm anterior to the lambda suture. The impact was then delivered at 2 mm depth (velocity 5 m/s, dwell time 100 ms) to produce a moderate level of injury. All animals then received a loose fitting 7 mm plastic cap secured over the craniectomy with Vetbond (3 M, St. Paul, MN). The skin was closed with interrupted sutures and treated with antibiotic ointment before removing the mouse from anesthesia and allowing recovery on a warming pad. One day after surgery, animals who had undergone CCI experienced hypoxemia (8% O_2_, 4% CO_2_) for 60 min in a Coy labs hypoxia chamber (Coy Laboratory, Grass Lake, MI). A mixture of N_2_, O_2_, and CO_2_ was utilized to maintain normocarbic hypoxemia [31]. Sham animals did not experience hypoxemia and were placed in cages directly next to the chamber while injured animals were in hypoxia chamber. After hypoxemia, sham and CCI littermates were returned to their original cages. All animals were subjected to identical transport and handling regardless of group assignment throughout all experiments.

### Minocycline administration

Previous preclinical studies have reported efficacy of minocycline for brain injury with dosing ranging from 22.5 to 90 mg/kg/dose [19, 24, 32, 49]. We designed a two-step funnel design for four different dosing regimens of minocycline with an initial evaluation of efficacy using short-term neuropathology followed by selecting the most efficacious dosing for long-term behavioral outcome studies. On post injury day 1, 2 h prior to hypoxemia, animals were randomized to receive minocycline (Sigma, St Louis, MO) 45 mg/kg, 90 mg/kg, 180 mg/kg or saline vehicle via intraperitoneal (i.p.) injection. Minocycline was dissolved in sterile saline at a concentration of 12.5 mg/mL in a water bath heated to 80 °C and then sterile filtered through a 0.22 µm filter. Control animals received an equal volume of sterile saline. First injections were administered to injured and sham animals 22–25 h after surgery. For 3 of the minocycline groups, the respective doses were repeated on post injury days 2 and 3. For the fourth minocycline group, after receiving an initial dose of minocycline 90 mg/kg 2 h prior to hypoxemia, mice received an additional 5 doses of minocycline 45 mg/kg every 12 h. Because of the variation in number and volume of i.p. injections, each minocycline group had their own saline vehicle-control group for short-term neuropathological analysis.

### Immunohistochemistry

Mice were euthanized under isoflurane anesthesia by transcardial perfusion with cold 0.3% heparin in PBS followed by 4% paraformaldehyde (PFA) (Sigma, St. Louis, MO). Whole brains were removed and post-fixed in 4% for 24 h, followed by equilibration in 30% sucrose for at least 48 h before sectioning. Serial 50-µm thick coronal slices were cut on a freezing microtome starting with the appearance of a complete corpus callosum (CC) and caudally to bregma − 3.08 mm. Immunohistochemical staining was performed on free-floating sections washed in Tris-buffered saline (TBS) between applications of primary and secondary antibodies. Endogenous peroxidase was blocked by incubating the tissue in TBS plus 0.3% hydrogen peroxide for 10 min. Normal goat serum (3%) in TBS with 0.25% Triton X (TBS-X) was used to block nonspecific staining for all antibodies. Slices were then incubated at 4 °C overnight with the primary antibodies (Table 1). For colorimetric immunohistochemistry, antibody binding was detected by incubating sections with biotinylated secondary antibodies (Table 1) in TBS-X Colorization was achieved using the VECTASTAIN Elite avidin–biotin complex (ABC)-HRP kit solution (Vector Laboratories, Burlingame, CA) followed by the application of 3–3′-diaminobenzidine (3–3′-DAB, Sigma-Aldrich). Sections were mounted on glass slides in TBS-X, dried and dehydrated in 50%, 70%, 95% and twice with 100% ethanol followed by Xylene (Sigma-Aldrich) before coverslipping with Dibutylphthalate Polystyrene Xylene (DPX, Sigma-Aldrich). For fluorescent immunohistochemistry, antibody binding was detected by incubating sections with Alexa fluorescence secondary antibody (Table 1) for 2 h. Sections were mounted on glass slides in TBS-X, dried, and coverslipped with mounting medium for fluorescence with DAPI.

**Table 1:** Overview of the primary antibodies used in the present study

Antibody	Fluorophore	Clone	Species	Dilution		Source	Product number
				Tissue	In vitro		
				IHQ	FC		
CD45	BV425	30-F11	Rat monoclonal		1:200	BioLegend	103134
CD3ɛ	AF700	500-A2	Armenian Hamster monoclonal		1:100	BioLegend	100320
CD11b	BV510	M1/70	Rat monoclonal		1:500	BioLegend	101263
MHC-II	PerCP-710	AF6-120.1	Mouse monoclonal		1:200	eBioscience	46-5320-80
TLR4	PE-Cy7	SA15-21	Rat monoclonal		1:250	BioLegend	145407
Ly6C	BV785	HK1.4	Rat monoclonal		1:2000	BioLegend	128041
Ly6G	AF700	1A8	Rat monoclonal		1:100	BioLegend	127622
NeuN		A60	Rabbit polyclonal	1:4000		Millipore	MAB377
Iba1		NCNP24	Rabbit polyclonal	1:1000		Wako	019-19741
CD68		Gp110	Rabbit polyclonal	1:1000		Invitrogen	PA5-32330
PDGFR-α			Goat polyclonal	1:250		R & D systems	AF1062
PSD-95			Rabbit polyclonal	1:200		Invitrogen	51-6900
Synapsin			Guinea Pig	1:1000		Synaptic systems	106 004
Secondary antibody	AF488		Goat anti-rabbit	1:500		Thermo Fisher	A-32731
Secondary antibody	AF568		Goat anti-guinea pig	1:500		Thermo Fisher	A-11073
Secondary antibody	AF568		Donkey anti-goat	1:500		Thermo Fisher	A-11055
Secondary antibody	AF488		Donkey anti-rabbit	1:500		Thermo Fisher	A-21206
Secondary antibody			Biotinylated goat anti-rabbit	1:1000		Vector Laboratories	BA-1000-1.5

### Quantification of immunohistochemistry

Stereological analysis was performed using StereoInvestigator software (MBF Bioscience, Williston, Vermont). Assessments were made by an investigator blinded to group assignment. The optical fractionator function was used to quantify target markers per cubic millimeter of tissue. A grid size of 125 × 125 µm and a counting frame of 25 × 25 µm was used for stereological quantification of NeuN+ cells in the pyramidal layer of Cornu Ammonis (CA) 3 region of the ipsilateral hippocampus. For stereological quantification of Iba1+ or CD68+ cells in the CA3 region of the ipsilateral hippocampus, the optical fractionator function was again used, with a grid size of 180 × 180 µm and a counting frame of 80 × 80 µm. For all stereological quantifications 4 slices spaced 300 µm apart were analyzed. The volume of the region of interest was calculated using the Cavalieri estimator. Gunderson’s coefficients of error were < 0.1 for all stereological quantifications.

### Three-dimensional reconstruction of microglia and oligodendrocyte progenitor cells

Microglia (Iba1) and oligodendrocyte progenitor cells (OPC, PDGFR-α) morphology analysis was performed as previously described [17]. Fifty-μm sections were stained with Iba1 and PDGFR-α at 4 °C overnight (Table 1), followed by Alexa Fluor conjugated secondary antibody staining (Table 1) for 2 h. Sections were mounted on glass slides in TBS-X, dried, and coverslipped with mounting medium for fluorescence with DAPI. Imaging was performed on a Zeiss LSM 880 confocal laser scanning microscope (Zeiss, White Plains, NY) using a 20× 0.8 NA objective. Z-stacks were done with 1.00-μm steps in z direction; 1024 × 1024 pixel resolution were recorded and analyzed using IMARIS software (Bitplane, Concord, MA). For microglia 3D reconstruction, a total of three hippocampal Iba1+ cells from the CA3 region of the hippocampus from one slice were analyzed by an investigator blinded to group assignment. For OPC 3D reconstruction, a total of 9 PDGFR-α + cells from the hilus of the ipsilateral dentate gyrus (DG) from 3 slices were analyzed (three PDGFR-α + cells per slice) by an investigator blinded to group assignment.

### Fluorescence immunohistochemistry and quantification

To perform quantitative analysis of OPC density in the DG region of the hippocampus, fluorescent images were obtained with a Zeiss Axio Imager Z2 with Apotome 2 with a 20× objective. The region of interest was the hilus of the DG. Quantification was performed on 3 coronal slices per mouse spaced 300 µm apart.

### Cresyl violet-staining

Cresyl violet staining was used for the detection of Nissl bodies in the cytoplasm of neurons on PFA sections in order to measure lesion and hippocampal volume. Twelve 50-µm thick slices sampled at 300 µm intervals starting with the appearance of the CC were utilized. After 3 washes in TBS, tissue was mounted on charged slides and dried overnight. The following day, slides were put in a cylinder glass holder and incubated in FD cresyl violet solution (FD Neurothecnologies, Inc, Colombia, MD) for 10 min. The remaining cresyl violet solution was rinsed away with water for 20 min. Then, the slides were dried and dehydrated in 95% ethanol (10 min), twice in 100% ethanol placed in xylene (8 min) before being coverslipped with DPX.

### Lesion volume analysis

The extent of tissue loss in the ipsilateral hemisphere for each animal was quantified using images of cresyl violet-stained slices acquired at 5× objective with a Zeiss Axio Scan Z1 Brightfield microscope (Zeiss, White Plains, NY). Tissue loss in the injured hemisphere was calculated as a percentage of the tissue volume in the contralateral hemisphere as described by others [47].

### Myelin Black Gold II staining

Myelin Black Gold II (BGII, Histo-Chem, Jefferson, AR) staining was used for visualizing individual myelin fibers in the CC in order to assess white matter injury. After 3 washes in TBS, free floating slices were incubated for 12 min and 60 °C in pre-warmed BGII solution (0.3% in 0.9% NaCl), followed by 2 washes in distilled water. Slices were fixed in pre-heated sodium thiosulfate (1% in distilled water) at 60 °C for 3 min. Tissue was mounted on charged slides and dried overnight. The following day, slides were dehydrated using a serial of graded alcohols (50%, 70%, 95% and twice with 100%) and coverslipped with DPX. Slides were scanned at 10× objective with a Zeiss Axio Scan Z1 Brightfield microscope (Zeiss, White Plains, NY). Four slices spaced 300 µm apart were analyzed with the most rostral slice being the first appearance of the dorsal hippocampus. Myelin percent area in the CC was quantified using ImageJ software. The CC region of interest was defined as the white matter area between the bilateral edges of the cingulum.

### Cell suspensions

The blood and the ipsilateral cortex and hippocampus were taken 7 days after CCI or sham surgery. Mice were anesthetized with isoflurane, and blood samples were taken in EDTA tubes immediately before transcardial perfusion with ice-cold 0.1 M heparinized-PBS. The brain regions of interest were dissected out on ice and digested at 37 °C for 15 min with collagenase D (400 units/mL, Roche) in Dulbecco’s PBS (Lonza, Basel, Switzerland), each containing 50 μg/mL of DNase I (Sigma-Aldrich). The tissue was then mechanically dissociated with a glass Pasteur pipette, filtered through a 70-μm nylon cell strainer, and centrifuged at 950 rpm for 15 min. A 25% Percoll (Sigma-Aldrich) column was used to remove cell debris and myelin, followed by centrifugation at 1700 rpm for 10 min. 50 µl-blood sample was mixed with 1 × Red Blood Lysis Buffer (Roche) and incubated in rotation for 15 min at room temperature (RT). Samples were then centrifuged at 3500 rpm for 5 min at RT. The supernatant was discarded, and cells were washed and resuspended in 1 mL of cytometer buffer [0.5% bovine serum albumin (Sigma-Aldrich), 5 mM EDTA (Millipore, Burlington, MA) in PBS]. The cells were resuspended in 100 µl of cytometer buffer and stained.

### Flow cytometry analysis

Cells were incubated for 5 min at RT with Zombie NIR Dye (BioLegend, San Diego, CA, USA) to assess their viability. The Zombie NIR Dye was quenched, and cells were washed with cytometry buffer and blocked with FcR blocking reagent (1:50, Miltenyi Biotec, Bergisch Gladbach, Germany). Then, the samples were washed with cytometry buffer, stained with antibodies (Table 1) for 15 min at RT, and analyzed on a BD LSRFortessa flow cytometer (BD Biosciences, Franklin Lakes, NJ) using the Software v10.6.1 (BD Biosciences, Franklin Lakes, NJ). Microglial cells were defined as CD45^low^CD11b^+^ and T cells as CD45^high^CD11b^−^CD3^+^.

### Quantification of synaptic loci

Quantification of synaptic loci was performed utilizing a semi-automated pipeline based on MATLAB (MathWorks, Portola Valley, CA) and Imaris 9.3.1 software (Bitplane, Concord, MA) as previously described [36]. Three confocal images were obtained on a LSM 880 microscope with AiryScan detector (Zeiss) from the ipsilateral molecular layer of the DG for each animal. Spots were detected for each channel using an x–y size of 0.2 μm, a z size of 0.6 μm, and automated background subtraction. A 0.1-μm x–y and 0.3-μm z guard was applied to exclude spots intersecting the edge of the image volume. Synaptic loci were identified using previously developed MATLAB scripts to find the nearest neighbor based on the x–y–z centroid of the top 20% brightest puncta. A cutoff of 260 nm pre-to-postsynaptic separation was used to quantify synaptic loci.

### Behavioral studies

Animals underwent behavioral testing 6 months after sham surgery or CCI. A total of 39 male and 39 female mice underwent long-term behavioral testing divided into six same sex cohorts of 13–15 animals. In each cohort, animals were randomized to sham surgery, injured-mice treated with vehicle (CCI-vehicle), or injured-mice treated with minocycline (CCI-minocycline). On post-injury day 1, injured animals were randomized to receive minocycline every 12 h (first dose 90 mg/kg, followed by 5 doses of 45 mg/kg i.p.) or an equivalent volume of normal saline. All tests were conducted by an experimenter blinded to group assignment. Animals were handled by the experimenter on three consecutive days prior to the initiation of testing. The order of tests was as follows: novel object recognition (NOR), followed by fear conditioning with each test performed 2 weeks apart. Prior to each testing day, animals were acclimated to the testing room for 1 h.

### Novel object recognition test

NOR testing was performed as previously described [8, 27]. Briefly, mice were handled by the experimenter twice a day 2 days before the beginning of the behavioral test. For the performance of the test, we used a square 4-chamber open field apparatus made of grey durable Plexiglas material (40 × 40 × 30 cm), and the luminosity of the room was adjusted with a luxmeter to obtain a light intensity of 20 lx in the center of the open field box. Animal activity was recorded automatically by a SMART video tracking software (Panlab Harvard apparatus, Barcelona, Spain) using an overhead USB-camera (Logitech, Newark, CA). On day 1 and 2 (habituation phase), mice were placed in the different arenas and allowed to explore the space for 5 min per day. On day 3 (familiarization session), mice were placed in the open field box in contact with two identical objects (towers of Lego bricks) at 5 cm from the walls for 10 min. On day 4 (test day), mice were returned to the arenas where one of the objects was changed for a new object (small falcon tissue culture flask half-filled with mouse bedding). On day 1 and 2, time in center, time in periphery, and total distance were analyzed. We calculated the Discrimination Index (DI), allowing discrimination between the novel and familiar objects, i.e., the exploration time for novel object (TN) was divided by the total amount of time interacting with the novel and familiar objects (TF): %DI = (100 × TN)/(TN + TF). After every session, the open field box and objects were cleaned with 70% ethanol to minimize olfactory cues.

### Contextual and cued-fear conditioning

Fear conditioning was performed 2 weeks after NOR as previously described [8, 9]. A fear conditioning-system (Ugo Basile, Gemonio, Italy) consisting of a sound-attenuating box (48.5 × 38.5 × 48.5 cm) with ventilating fan, a light, overhead USB-camera (Logitech), and an electrical grid floor for inducing the foot-shocks was utilized. On day 1 (conditioning), mice were placed in context A and every 1.5 min they received 5 tone-shock pairings [30 s (s) tone with 0.5 mA and shock during the last 2 s] and the freezing time in 30 s epochs was measured by a blinded observer. On day 2 (contextual test), mice were placed in context A for 10.5 min and freezing time was measured to assess contextual fear memory. On day 3 (cued test), mice were placed in a novel context B (checkered walls and white hard cover on the floor) to eliminate any confounding interactions of contextual fear for 10.5 min and subsequently given five 30 s tones without any shocks. On day 1 and 3, after the final tone-shock pairing, mice remained in the conditioning chamber for 30 s before being returned to their home cages. Freezing was defined as the absence of visible movement except that required for respiration. Percentage of total freezing time was calculated by dividing the amount of time-spent freezing by total time (630 s).

### Statistical analysis

All data for each animal was entered and tracked utilizing a REDCap database to maintain data integrity [21]. For initial short-term studies, experiments were powered to detect a 30% difference between CCI-vehicle and CCI-minocycline treated animals. For long-term behavior studies, experiments were powered to detect a 33% improvement in performance of minocycline treated mice compared with vehicle controls. Data was assessed for normal distribution with Shapiro Wilk test. Student *t* test or Mann Whitney U test were used for histological data and behavioral data when appropriate. For long-term behavior studies, experiments were conducted in cohorts of 10–15 animals. Before combining cohorts, two-way ANOVA was performed with cohort and group to confirm that cohort was not a significant factor or had a significant interaction with group. ANOVA and Kruskal–Wallis test were employed for normally and non-normally distributed data respectively. For sex dependent differences a two-way ANOVA was utilized. All analysis was performed with Statistica v13.3 (TIBCO software. Palo Alto, CA).

## Results

### Minocycline administration prior to secondary hypoxemia provides short-term neuroprotection

**Fig. 1:**
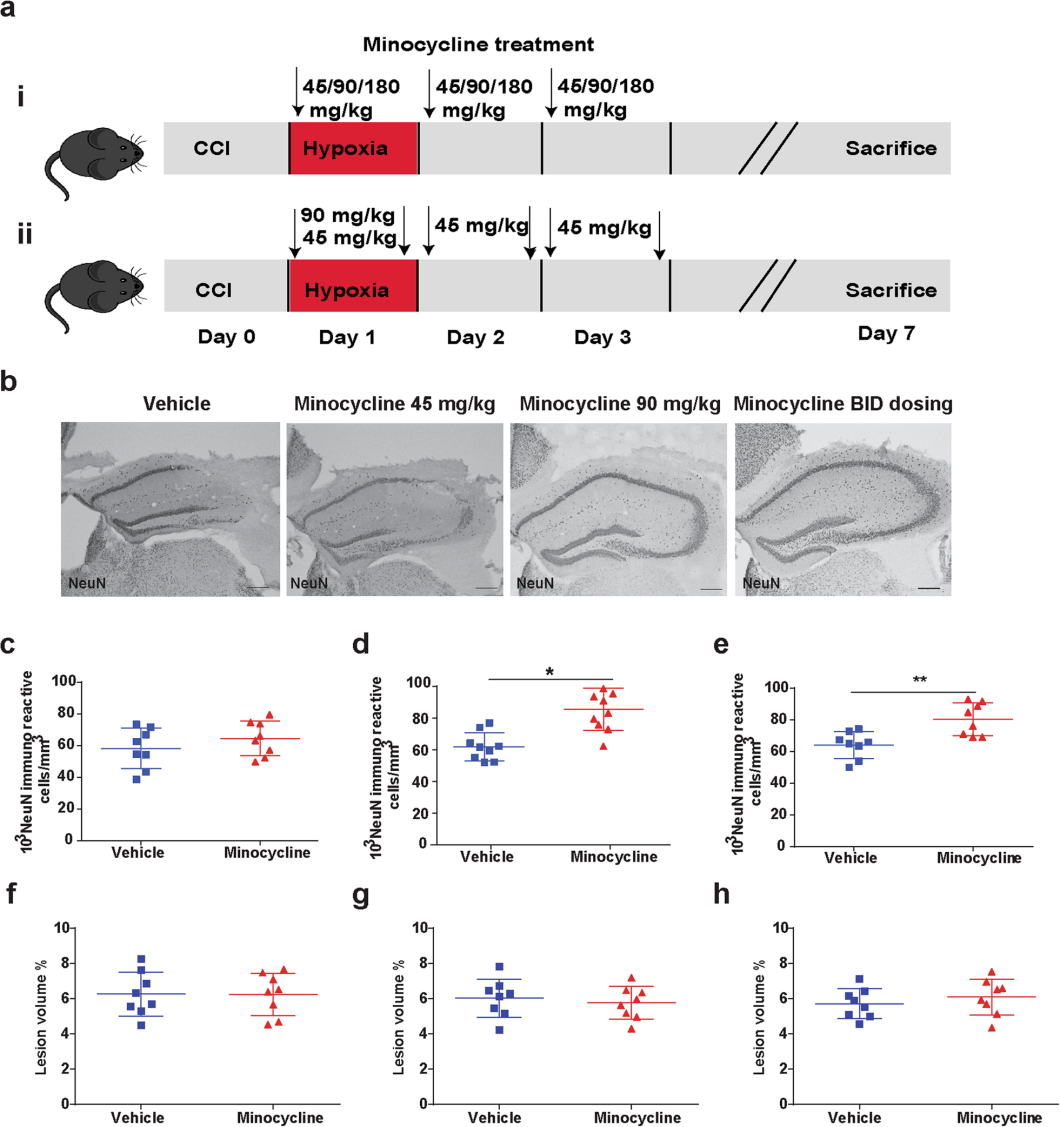
Minocycline administration prior to secondary hypoxemia provides short-term neuroprotection. **a** Experiment designs. **b** Representative image of NeuN+ cells in CA3 region of injured hippocampi and **c** stereological quantification of 45 mg/kg dosage, **d** 90 mg/kg dosage and, **e** 90/45 mg/kg dosage. **f** lesion volume of 45 mg/kg dosage, **g** 90 mg/kg dosage and, **h** 90/45 mg/kg dosage. Mean values are plotted ± SD, unpaired *t* test **p < 0.01, n = 8–9 mice per group

In our initial short-term studies, we selected 4 different dosing regimens of minocycline (Fig. 1ai, ii). Minocycline dosing of 180 mg/kg resulted in 80% mortality in the minocycline group by post-injury day 3 and no further investigation with this dose was performed. For our initial assessment of efficacy, we quantified neurons in the CA3 region of the hippocampus (Fig. 1b), a population known to be especially vulnerable to death post-CCI [11]. Minocycline dosing at 45 mg/kg once a day did not show evidence of neuronal protection (Fig. 1c). However, both dosing regimens with an initial dose of 90 mg/kg demonstrated similar reduction in neuronal loss (Fig. 1d, e). Based on the pharmacokinetics of minocycline in mouse models, we proceeded to utilize the twice a day dosing regimen (Fig. 1aii) for further study which minimizes peak plasma concentrations and more closely emulates human dosing of minocycline [41]. We did not observe any differences in lesion volume at 1 week post injury between any of the groups (Fig. 1f–h).

### Minocycline modulates microglial activation after delayed hypoxemia and TBI

**Fig. 2:**
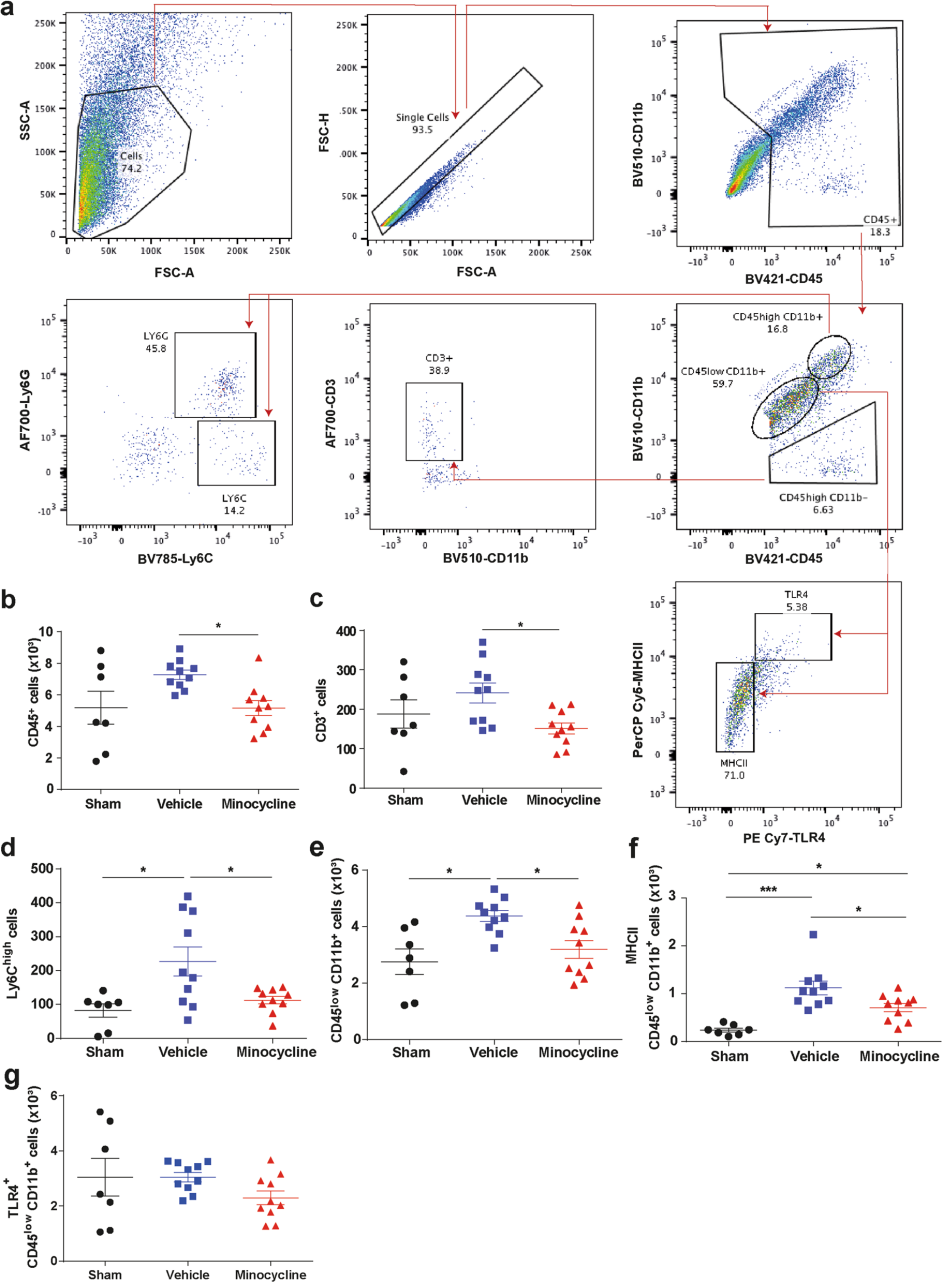
Minocycline alters the immune system response in the hippocampus and cortex. **a** Gating strategy. **b**–**g** Quantification of absolute numbers in the ipsilateral hippocampus and cortex. **b** CD45^+^ cells, **c** CD3^+^ cells, **d** monocytes **(**Ly6C^high^), **e** microglia (CD45^low^CD11b^+^), **f** MHCII^+^ microglia, and **g** TLR4^+^ microglia. Mean values are plotted ± SD, One-way ANOVA followed by Tukey multiple comparison post hoc test were used to determine statistical differences; *p < 0.05. n = 8–10 per group. Major histocompatibility complex II (MHCII). Toll-like receptor 4 (TLR4)

Minocycline when administered within 1 h after experimental TBI has been reported to suppress microglial activation [42]. To assess the impact of minocycline on the neuroinflammatory response after TBI and delayed hypoxemia, we performed flow cytometry on the ipsilateral cortex and hippocampus 7 days after injury (Fig. 2a). We observed a reduction in the total number of microglia (CD45^low^CD11b^+^, Fig. 2b) and the number of microglia expressing major histocompatibility complex II (MHCII) minocycline treated animals (Fig. 2c). Additionally, we found reduced brain infiltration of peripheral lymphocytes (CD3^+^ Fig. 2e) and monocytes (Ly6C^+^, Fig. 2f) in CCI-minocycline compared with CCI-vehicle. Flow cytometry of peripheral blood samples taken at time of sacrifice from the same mice revealed no differences in the peripheral blood immune cell populations arguing against systemic suppression of the peripheral immune response by minocycline (Additional file 1: Figure S1).

**Fig. 3:**
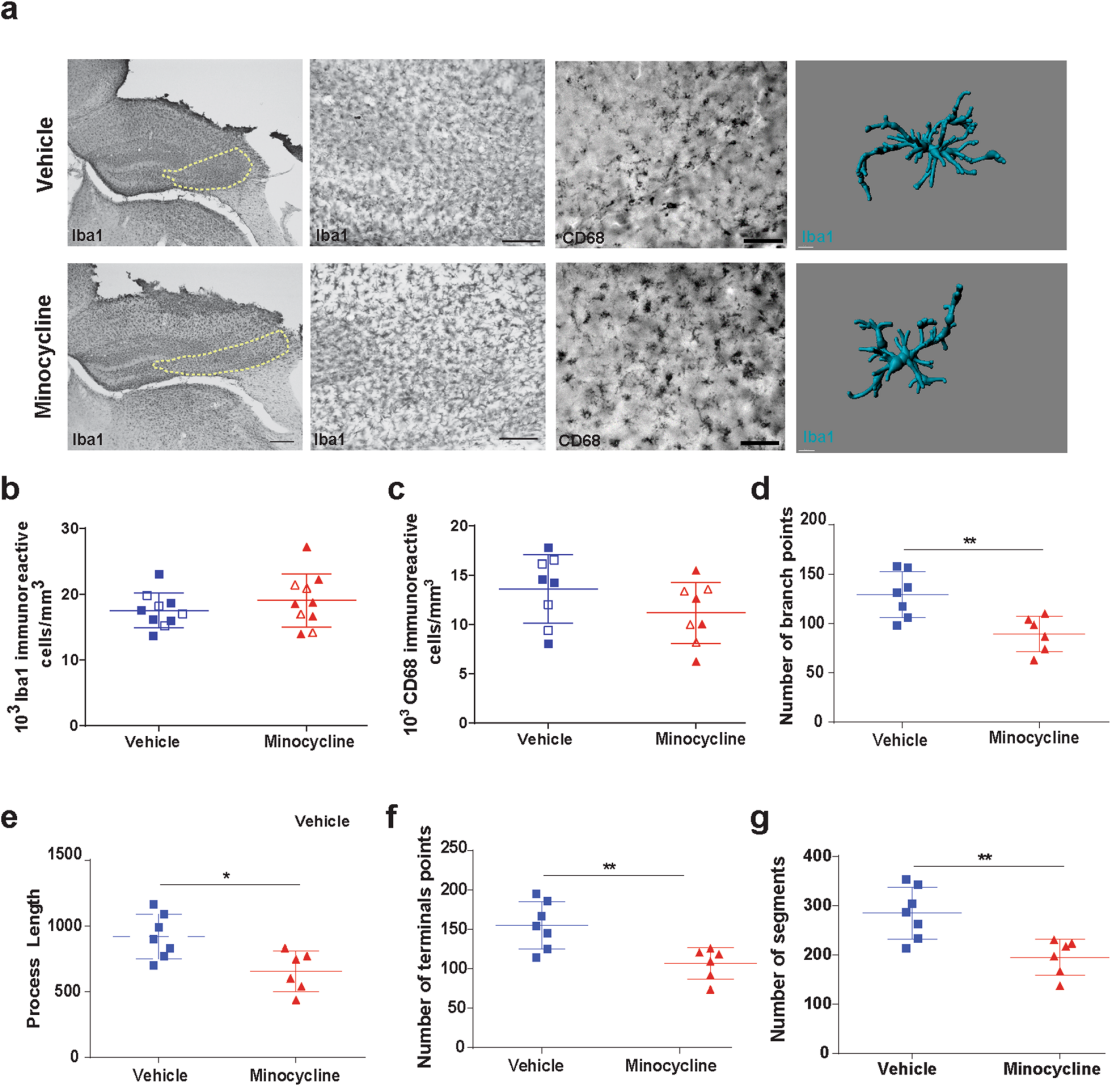
Minocycline modulates microglia morphology. **a** Representative image of Iba1+ and CD68 + cells in CA3 region of injured hippocampi. Region of interest for stereological quantification represented by yellow dashed line. Representative three-dimensional reconstruction images of microglia cells. **b** Quantification of the Iba1+ cells density. **c** Quantification of the CD68+ cells density. Male mice represented by closed symbols, female mice represented by open symbols. Quantification of the microglia morphology of **d** number of branch points, **e** process length, **f** number of terminal points, and **g** number of segments. Mean values are plotted ± SD, unpaired *t* test **p < 0.01, n = 8 mice per group

We next performed traditional stereological quantification of Iba1+ cells in the CA3 region of the hippocampus. No differences in the number of Iba1  microglia was found between vehicle and minocycline treated mice at 1-week post injury (Fig. 3a, b). We also did not observe any sex dependent effects (data not shown). We further attempted to discern differences in activated microglia by utilizing the lysosomal marker CD68, but again stereological quantification did not reveal any significant differences between vehicle and minocycline treated injured mice in the CA3 region of the hippocampus (Fig. 3a, c). Minocycline has been reported to alter microglial morphology towards a more ameboid morphology in a perinatal mouse model [45]. To further characterize microglial changes with minocycline administration, we performed semi-automatic quantitative morphometric three-dimensional measurements of hippocampal microglia [17]. At 1 week post injury, hippocampal microglia from CCI-minocycline had decreased branch points (Fig. 3d), process length (Fig. 3e), terminal points (Fig. 3f), and number of segments (Fig. 3g) compared with CCI-vehicle. These data support minocycline’s modulation of microglial activity and neuroinflammation as its mechanism for neuroprotection.

### Minocycline does not improve acute post-TBI myelination but alters oligodendrocyte-progenitor cell morphology

**Fig. 4:**
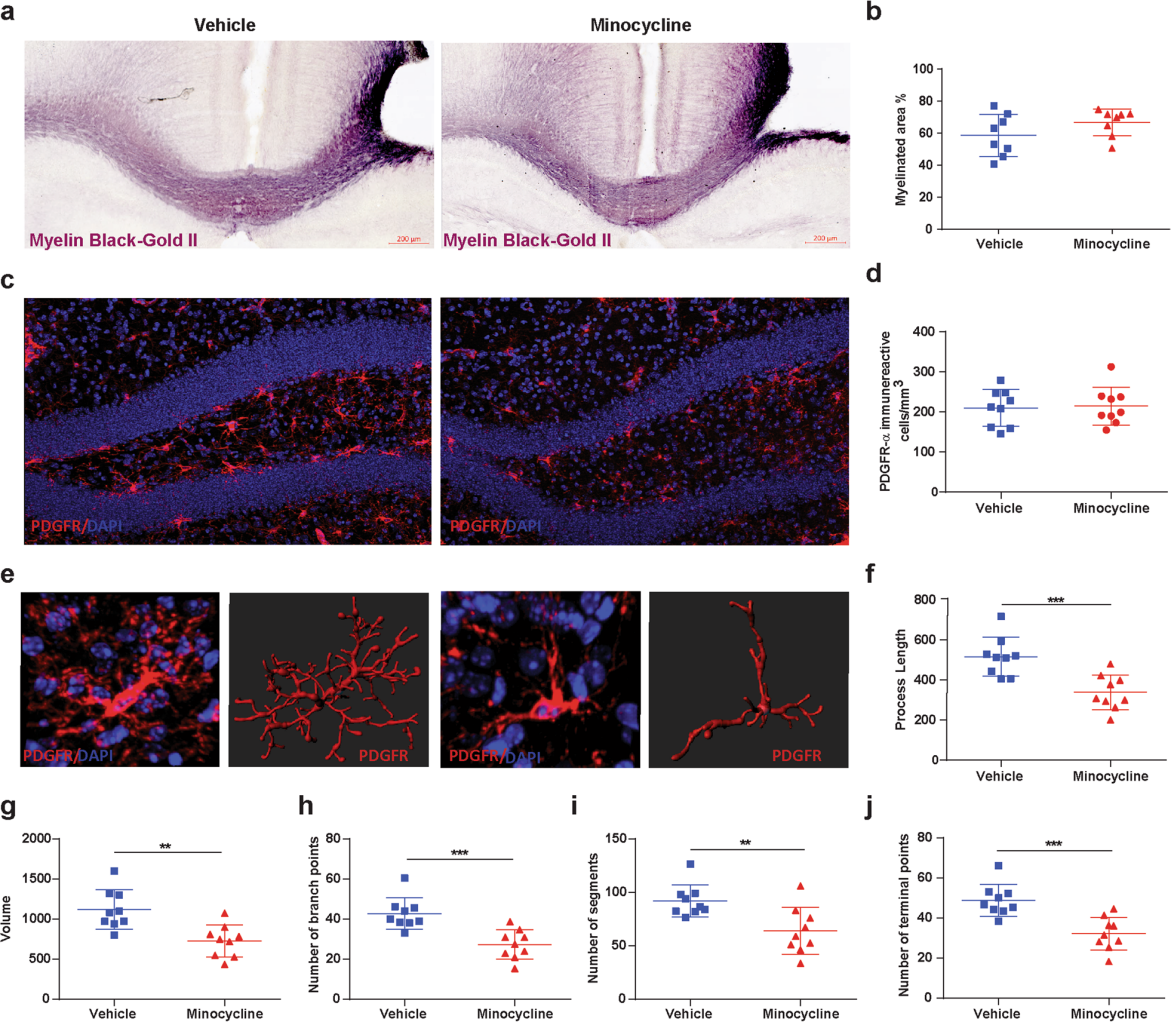
Minocycline does not improve acute post-TBI myelination but alters OPC morphology. **a** Representative image of myelin black gold staining and **b** quantification of percentage of myelinated area. **c** Fluorescent image of PDGFR-α in DG and **d** quantification of PDGFR-α + cells density. **e** Representative three-dimensional reconstruction images of PDGFR-α + cells. **f**–**j** Quantification of the OPC morphology. **f** process length, **g** volume, **h** number of branch points, **i** number of segments and, **j** number of terminal points**.** Mean values are plotted ± SD, unpaired *t* test **p < 0.01, n = 8 mice per group

Previously we have reported, in our mice model of TBI with delayed hypoxemia, extensive white matter injury in the peri-contusional CC [31]. To assess the impact of minocycline administration on white matter injury after CCI, we performed an analysis of BGII staining of the myelin fibers in the peri-contusional CC at 1 week after injury (Fig. 4a). Minocycline treatment had no effect on the percentage of myelinated area in the CC 1 week after injury (Fig. 4a, b).

Microglial activation after TBI contributes to T cell infiltration and impacting mature oligodendrocyte and OPC cell response [40]. We performed a quantitative analysis of PDGFR-α + OPCs at 1-week post injury in the DG region of the ipsilateral hippocampus (Fig. 4c). Fluorescent immunohistochemical analysis of OPCs density revealed no significant difference between vehicle and minocycline treated animals (Fig. 4d). We then performed an analysis of OPC morphology in the DG at the same time point (Fig. 4e). Minocycline treatment resulted in significant decreases in process length (Fig. 4f), volume (Fig. 4g), number of branch points (Fig. 4h), number of segments (Fig. 4i), and terminal points (Fig. 4j) compared with vehicle. Suggesting that modulation of microglial activation after injury influences oligodendrogenesis.

### Minocycline improves long-term contextual-fear memory performance

**Fig. 5:**
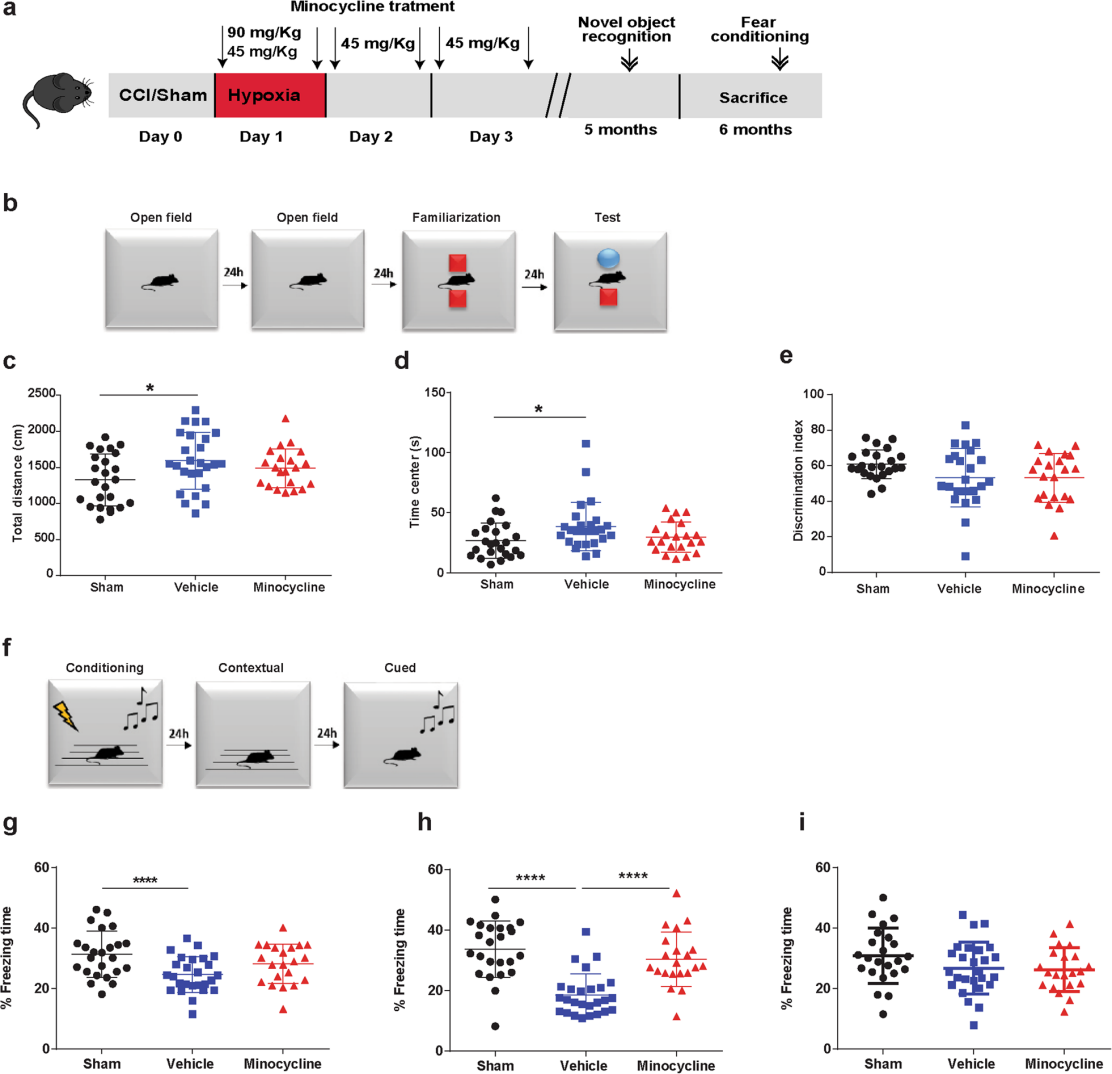
Minocycline impacts long-term behavior. **a** Experiment design. **b** Novel object recognition paradigm. **c** Total distance, F_(2, 68)_ = 3.591, *p* = 0.033. **d** Time in the center F_(2, 68)_ = 3.583, *p* = 0.033 and, **e** discrimination index. **f** Fear conditioning paradigm. Percentage of freezing time **g** on day 1 or conditioning F_(2, 68)_ = 6.289, *p* = 0.003, **h** on day 2 or contextual test F_(2, 68)_ = 21.87, *p* < 0.0001, and **i** on day 3 or cued test. Mean values are plotted ± SD, one-way ANOVA followed by Tukey multiple comparison post hoc test were used to determine statistical differences; *p < 0.05, ****p < 0.0001. n = 21–24 per group

We next performed a long-term preclinical trial of our selected minocycline dosing (initial 90 mg/kg followed by 5 doses of 45 mg/kg every 12 h) (Fig. 5a). 76 mice (38 male and 38 female) were randomized sham or CCI-vehicle or CCI-minocycline. Five mice (all female) did not survive to behavior testing. One mouse randomized to minocycline was sacrificed 4 h after CCI due to an inability to return to general husbandry care. Four other mice died within seven days of surgery (2 sham and 2 minocycline mice). During open field testing (Fig. 5b), CCI-vehicle were more active (Fig. 5c) and spent more time in the center region (Fig. 5d) compared with sham. CCI-minocycline were not statistically different than sham or CCI-vehicle on post-hoc Tukey. Sham animals had better performance in NOR compared with injured animals but did not reach statistical significance F_(2,70)_ = 3.87, *p* = 0.09 (Fig. 5e).

Changes in the fear memory response have been associated with disruptions in the hippocampal-amygdala circuit in chronic TBI linked with post-traumatic stress disorder [3, 33]. We employed a 3-day fear memory paradigm with conditioning on day 1, contextual-fear response on day 2, and cued-fear response on day 3 (Fig. 5f). Sham animals had increased freezing compared with both injured groups during conditioning on day 1 (Fig. 5g). Interestingly, contextual-fear response which is hippocampal dependent was significantly different F_(2,70)_ = 26.7, *p* < 0.00001 (Fig. 5h). CCI-minocycline had significantly higher freezing response compared with CCI-vehicle. Cued-fear response, amygdala-dependent task, was no different across the three groups (Fig. 5i). We did observe sex differences in contextual fear between sham animals but no sex dependent differences in either of the injury groups (Additional file 2: Figure S2). These data highlight that in a focal injury model, acute minocycline administration resulted in improved long-term hippocampal-dependent fear memory response.

### Acute minocycline administration reduces long-term neuronal and synapse loss

**Fig. 6:**
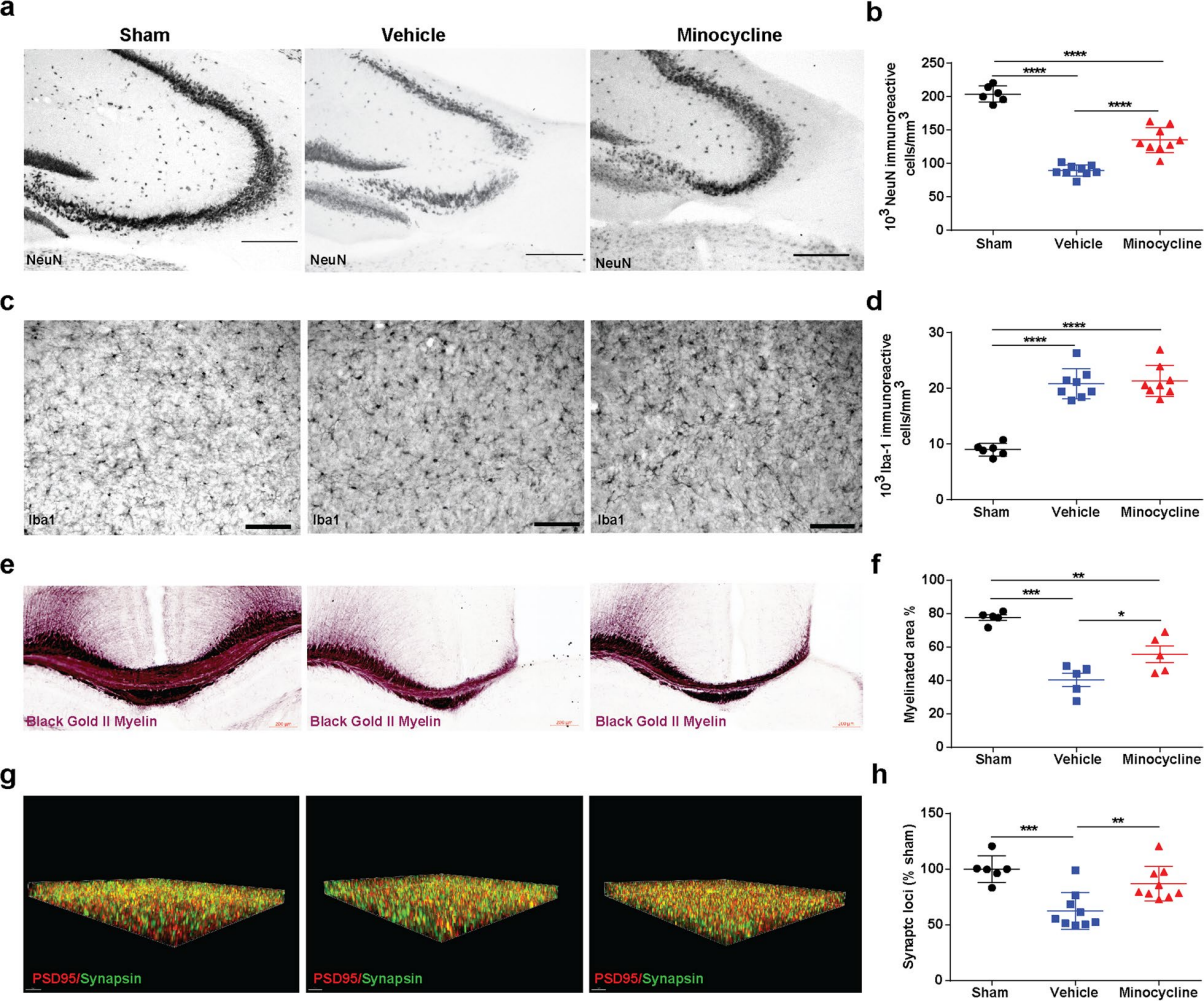
Acute minocycline administration reduces long-term neuronal and synapse loss. **a** Representative image of NeuN+ cells in CA3 region of injured hippocampi and **b** stereological quantification F(_2, 21_) = 117.7, *p* < 0.0001. **c** Representative image of Iba1 cells in CA3 region of injured hippocampi and **d** stereological quantification F(_2, 19_) = 52.5, *p* < 0.0001. **e** Representative image of myelin black gold staining and **f** quantification of percentage of myelinated area image of Iba1 cells in CA3 region of injured hippocampi and **d** stereological quantification F(_2, 12_) = 24.92, *p* < 0.0001. **g** Puncta detection of PSD95 (red) synapsin (green). **h** Quantification of synaptic loci (% sham) F(_2, 21_) = 12.14, *p* < 0.0003. One-way ANOVA followed by Tukey multiple comparison post hoc test were used to determine statistical differences; *p < 0.05. ***p < 0.01, ****p < 0.001, n = 5–9 per group

We next performed stereological quantification of the CA3 region of the hippocampus from brains of the mice that had undergone behavioral testing (Fig. 6a, b). We found reduced neuronal loss in CCI-minocycline compared with CCI-vehicle (Fig. 6b). Iba1 immunohistochemistry revealed increased chronic microglial activation in both injured groups compared with sham but no minocycline effect was observed (Fig. 6c, d). In addition, we also found that CCI-minocycline had increased myelinated area 6 month after injury compared with CCI-vehicle (Fig. 6e, f). Microglia play an important role in synaptic plasticity and memory [12]. Based on our findings of improved hippocampal dependent contextual fear memory response, we utilized a recently developed super-resolution analysis workflow, synaptic evaluation and quantification by imaging nanostructure (SEQUIN) to evaluate long-term changes in synaptic density in the molecular layer of the DG (Fig. 6g, h). We chose the molecular layer of the DG based on previous reports of changes in synaptic density in this region associated with neurodegenerative diseases, as well as changes induced by chronic stress and physical activity [18, 22, 37]. We found reduced number of synaptic loci in vehicle-injured mice compared with injured mice who had received acute administration of minocycline (Fig. 6h) suggesting that acute minocycline administration improves long-term synaptic density associated with behavioral improvements.

## Discussion

In summary, acute minocycline administration within a clinically feasible therapeutic window 24 h after experimental TBI and 2 h prior to systemic hypoxemia reduced microglial activation in the cortex and hippocampus and reduced acute neuronal loss in the CA3 region of the hippocampus. Furthermore, acute minocycline administration after injury improved fear memory response 6 months after injury and was associated with reduced hippocampal neuronal loss, reduced white matter myelin loss, and improved hippocampal synaptic density. Our results support the hypothesis that acute short-term administration of minocycline in a model of TBI with delayed systemic hypoxemia has a neuroprotective effect via reduced microglial activation.

Previous pre-clinical studies of minocycline in TBI have shown conflicting results on its neuroprotective efficacy [19, 35, 46]. These differences can be attributed to different injury models, variation in brain regions evaluated for changes in neuropathology, and timing/dosing of minocycline administration. To overcome some of these challenges we chose to first evaluate the short-term neuroprotective efficacy of various dosing regimens. Minocycline administered before injury or early after experimental TBI (within 30 min) has been shown to reduce neuronal death in focal open and closed head injury models [5, 35]. In our model of TBI, the CA3 region of the hippocampus has been shown to be highly susceptible to neuronal death [1]. We utilized stereological quantification of this region as our initial metric as minocycline has not shown to be effective at altering neurogenesis or reducing white matter injury at acute time points [19, 29]. The highest dosing regimen (180 mg/kg once a day) resulted in increased mortality compared with vehicle-injured mice. Mice receiving this higher dose of minocycline exhibited poor weight gain and acute impairments in gait and balance. Vestibular disturbances have been a reported side effect of minocycline use in humans and may account for the poor outcomes at this higher dose in our model [51]. We observed a dose–response in regards to neuroprotection in the CA3 region of the hippocampus with lower dosing (45 mg/kg/day) having no impact on neuroprotection and a robust response with dosing regimens of 90–135 mg/kg/day.

Minocycline has been shown to reduce microglial activation after CNS injury [7, 19]. We did not detect differences in microglia cells numbers utilizing traditional stereological quantification approaches with either Iba1 or CD68 immunohistochemistry in the ipsilateral hippocampus 1 week post-injury. In contrast, a flow cytometry approach of the ipsilateral hippocampus and cortex revealed minocycline’s anti-inflammatory properties with reduced number of microglia expressing MHCII. These differences found with orthogonal approaches could be attributed to stereological analysis being limited to the hippocampus and flow cytometry encompassing the entire injured cortex and hippocampus. We did not further explore changes in microglia phenotype populations in our studies. Future investigations utilizing advance techniques such as single cell RNA sequencing of microglia to further characterize minocycline induced changes in pro-inflammatory and anti-inflammatory microglia are planned.

Microglia, as the resident immune cells of the brain, are in part responsible for recruitment of peripheral immune cells to the site of injury [2, 4, 28]. Minocycline-treated mice had reduced T cell and monocyte infiltration into the hippocampus without any changes in peripheral blood immune cell numbers supporting the hypothesis that minocycline induced changes in the neuroinflammatory response are in large part due to reduced activation of microglia and subsequent reduced recruitment of peripheral immune cells to the brain. The role of the adaptive immune response in modulating the neuroinflammatory and thereby TBI severity and recovery remains unclear. Rag^−/−^ mice (absence of B and T cells) did not show any alteration in injury severity following CCI when evaluated 1 week after injury compared with wild type mice [50]. However, recent evidence has uncovered that the infiltration of lymphocytes peaks beyond the first week after TBI and subsets of T cells may have an impact on chronic neurodegeneration [13].

Timing and duration of minocycline administration in TBI may be a critical factor responsible for conflicting evidence for its neuroprotective efficacy in TBI. Seven days of minocycline administration after 3 repetitive head injuries in an immature rat model did reduce microglia activation but exacerbated cognitive deficits [19]. In a clinical trial of minocycline during the chronic phase of TBI demonstrated reduced microglial activation but elevations in plasma neurofilament light chain levels suggesting a detrimental effect of microglial activation on neurodegeneration in the chronic phase of TBI [39]. Pharmacologic depletion of microglia with PLX5622, CSF1R inhibitor, in a mouse model of TBI has demonstrated that the timing in relation to injury of microglial activation and repopulation has an impact on CNS pathology and recovery [52]. Our own findings of the protective/reparative effect of an acute short-term course of minocycline further highlight the temporal dependence of microglial manipulation’s influence on injury severity and recovery in TBI.

Minocycline administration at lower doses in hypoxic-ischemic (HI) models of brain injury have been shown to reduce HI-induced oligodendrocyte cell death and myelin loss [7]. However in a closed head injury model, acute administration of minocycline immediately after injury for 3 or 10 days did not reduce white matter injury as measured by β-amyloid precursor protein immunohistochemistry [20]. Microglia depletion prior to close head injury attenuated transcriptional changes in OPCs and myelinating oligodendrocytes after injury implicating an important role for microglia in white matter injury and recovery [53]. Our own studies did not find increased survival in OPCs with minocycline at 1 week post injury but we did observe changes in OPC morphology in minocycline-treated mice. These data were associated with long-term (6 months) reduction in myelin loss in minocycline–treated mice. One possible explanation for our findings is that OPC morphological changes in minocycline-treated mice at 1 week post-injury are associated with increased maturation of OPCs to become myelin generating mature oligodendrocytes resulting in remyelination of demyelinated axons. Future studies assessing the impact of minocycline on mature oligodendrocytes after injury as well as studies utilizing primary OPC and activated microglia co-cultured with minocycline to better understand the impact of these morphologic changes on proliferation, maturation and function are planned.

There are limitations in the experimental approach when translating our findings to the clinical setting. We utilized young adult mice of both sexes in our long-term survival studies, but did not explore the impact of age, which may have impact on the neuroprotective effects of minocycline. We utilized an open focal model of head injury which has the advantage of being reproducible reducing variation and animal’s number needed but does not encompass the full scope of TBI pathologies in the clinical setting. Our animal platform of TBI and delayed hypoxemia is modeled after the clinical scenario encountered by severe TBI patients in the intensive care setting [31]. All animals in our studies are required to return to general husbandry care limiting the severity of the TBI. We did not include an injury group without hypoxemia which limits our ability to untangle the effect of timing of minocycline administration in our model. Minocycline’s neuroprotective properties could simply related to a longer temporal window of efficacy than previously reported or it could be acting as a “pre-treatment” to reduce the deleterious impact of delayed hypoxemia. We administered our study drug i.p., whereas it would be expected to be given intravenously in the ICU setting reducing the translatability of the dosing regimens we employed. Furthermore, we did not perform pharmacokinetic studies determining minocycline brain concentrations in our injured mice.

Together, our data demonstrate that in a clinically relevant rodent model of TBI with secondary insult, acute minocycline administration modulates the neuroinflammatory response resulting in short- and long-term neuroprotection and improved fear memory performance at a long-term time point rarely assessed in preclinical models of TBI. Timing in relation to injury and duration of minocycline treatment and its impact on neuroinflammatory response may be responsible for extensive neuroprotection observed in our studies. Future investigations utilizing similar timing and dosing of minocycline in alternative experimental TBI models and species should be performed to confirm the efficacy of minocycline prior to conducting clinical investigations.

## Supplementary Information

**Additional file 1: Figure S1**. No immune system changes in the blood associated with acute minocycline administration after traumatic brain injury and delayed hypoxemia. a-d Peripheral immune system cell profile characterization by flow cytometry at 1 week after injury. Quantification of the absolute number of cells in the blood for a myeloid and lymphoid cells (CD45+), b CD3 Tcells (CD11b-CD3+), c monocytes (CD45highCD11b+Ly6C+), d neutrophils (CD45highCD11b+Ly6G+).

**Additional file 2: Figure S2**. Long-term behavior supplemental data. a Total distance and b total time in center divided by sex. c Total time spent with objects, Kruskal-Wallis test. p = 0.01. * p < 0.05, ** p < 0.01. d Discrimination index by sex. Fear conditioning e conditioning or day 1, f contextual fear or day 2 two-way ANOVA followed by post hoc tukey test. Group F(2, 65) = 59.44, p < 0.0001, Sex F(1, 65) = 6.684, p = 0.12, Sex*Group interaction F(2, 65)= 2.79, p = 0.069. * p < 0.05, g cued fear or day 3

## Data Availability

The datasets used and/or analyzed during the current study are available from the corresponding author on reasonable request.
