# Supplementary figures and images for: Acute minocycline administration reduces brain injury and improves long-term functional outcomes after delayed hypoxemia following traumatic brain injury
Source: Acta Neuropathol Commun. 2022 Jan 28;10:10. doi: 10.1186/s40478-022-01310-1 (PMC8796448; doi:10.1186/s40478-022-01310-1)

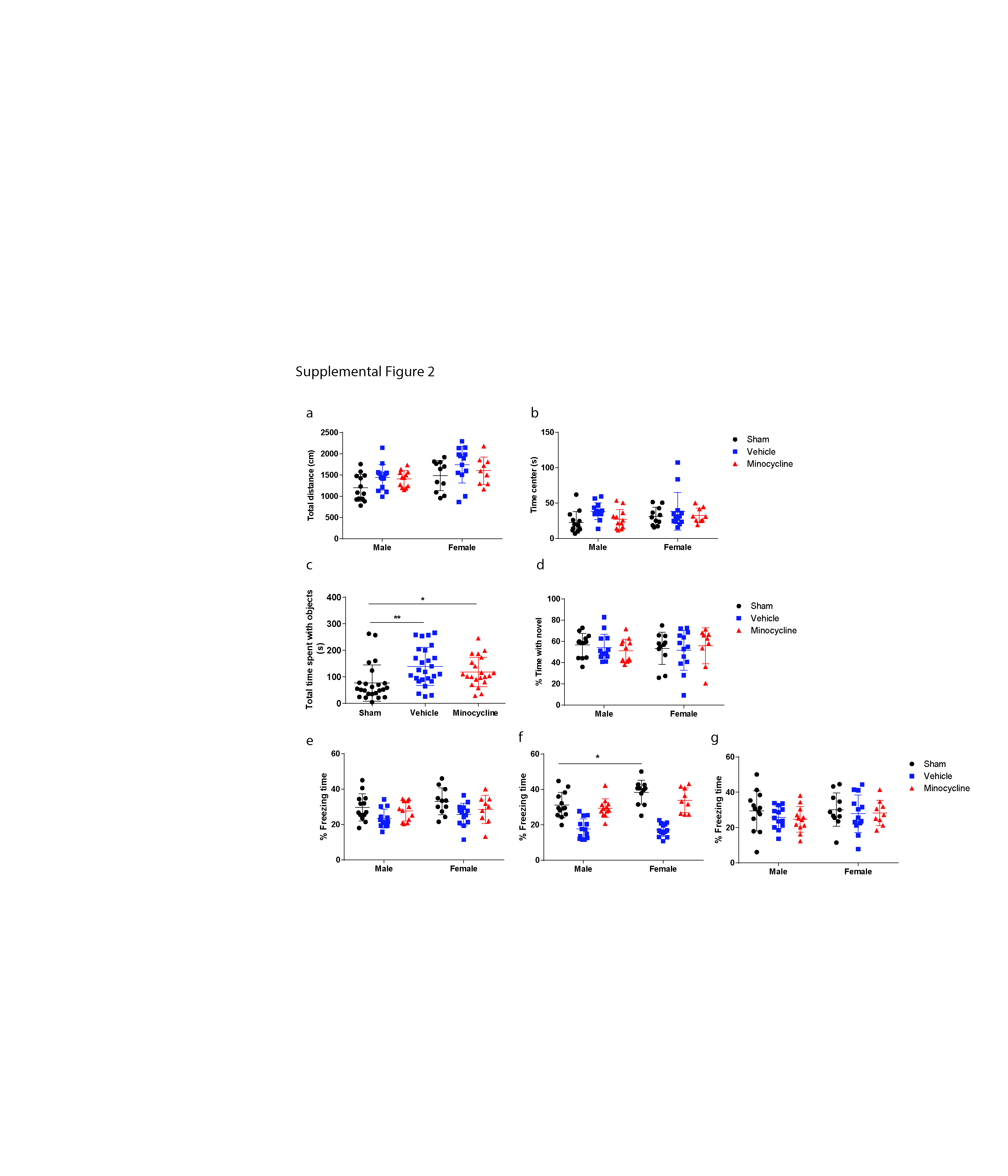

Supplement: Supplementary file 2 — Additional file 2: Figure S2. Long-term behavior supplemental data. a Total distance and b total time in center divided by sex. c Total time spent with objects, Kruskal-Wallis test. p = 0.01. * p < 0.05, ** p < 0.01. d Discrimination index by sex. Fear conditioning e conditioning or day 1, f contextual fear or day 2 two-way ANOVA followed by post hoc tukey test. Group F(2, 65) = 59.44, p < 0.0001, Sex F(1, 65) = 6.684, p = 0.12, Sex*Group interaction F(2, 65)= 2.79, p = 0.069. * p < 0.05, g cued fear or day 3 [file 40478_2022_1310_MOESM2_ESM.tif]
